# Mitochondrial phylogeny and phylogeography of East African squeaker catfishes (Siluriformes: *Synodontis*)

**DOI:** 10.1186/1471-2148-6-49

**Published:** 2006-06-19

**Authors:** Stephan Koblmüller, Christian Sturmbauer, Erik Verheyen, Axel Meyer, Walter Salzburger

**Affiliations:** 1 Department of Zoology, Karl-Franzens-University Graz, Universitätsplatz 2, 8010 Graz, Austria; 2 Vertebrate Department, Royal Belgian Institute of Natural Sciences, 1000 Brussels, Belgium; 3 Lehrstuhl für Zoologie und Evolutionsbiologie, Department of Biology, University of Konstanz, 78467 Konstanz, Germany

## Abstract

**Background:**

Squeaker catfishes (Pisces, Mochokidae, *Synodontis*) are widely distributed throughout Africa and inhabit a biogeographic range similar to that of the exceptionally diverse cichlid fishes, including the three East African Great Lakes and their surrounding rivers. Since squeaker catfishes also prefer the same types of habitats as many of the cichlid species, we hypothesized that the East African *Synodontis *species provide an excellent model group for comparative evolutionary and phylogeographic analyses.

**Results:**

Our analyses reveal the existence of six major lineages of *Synodontis *in East Africa that diversified about 20 MYA from a Central and/or West African ancestor. The six lineages show a clear geographic patterning. Two lineages are endemic to Lake Tanganyika (plus one non-endemic representative), and these are the only two *Synodontis *lineages that diversified further into a small array of species. One of these species is the cuckoo catfish (*S. multipunctatus*), a unique brood parasite of mouthbrooding haplochromine cichlids, which seems to have evolved in parallel with the radiation of its cichlid host lineage, the Tropheini. We also detect an accelerated rate of molecular evolution in *S. multipunctatus*, which might be the consequence of co-evolutionary dynamics.

**Conclusion:**

We conclude that the ancestral lineage of today's East African squeaker catfish fauna has colonized the area before the Great Lakes have formed. This ancestor diversified rapidly into at least six lineages that inhabit lakes and rivers in East Africa. Lake Tanganyika is the only lake harboring a small species flock of squeaker catfishes.

## Background

**Figure 1:**
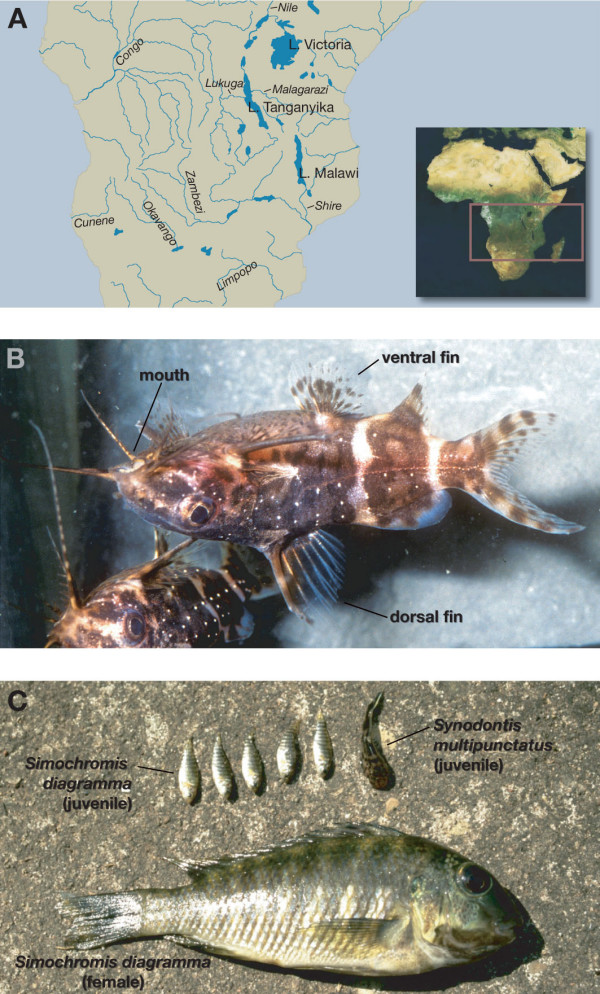
**The squeaker catfishes (*Synodontis*) in East Africa**. (*a*) Map of southern and eastern Africa showingthe Great Lakes in East Africa and the main river systems in thearea. (*b*) The upside-down catfish (*S. nigriventris*) is characterized by its inverse swimming posture. Photo courtesy of E. Schraml. (*c*) The cuckoo catfish(*S. multipunctatus*) from Lake Tanganyika is a brood parasiteof mouthbrooding haplochromine cichlids. Here, the mouth-content ofa breeding female of *Simochromis diagramma *is shown: Wefound five larvae of *S. diagramma *plus one larger cuckoocatfish larvae (Photo: W. Salzburger).

The Great Lakes in the East African Rift Valley (Fig. 1a) are home to an exceptionally diverse ichthyofauna [1-4]. The most famous elements of the lakes' faunas are the cichlid fishes that have formed species flocks of an unparalleled species-richness and degree of eco-morphological and behavioral complexity [1,5,6]. It has been estimated that almost 1,800 cichlid species inhabit Lakes Tanganyika, Malawi and Victoria [7,8] and that these lake endemic species are likely to have evolved in the last few millions or as recently as the last thousands of years only [9-12]. It is, thus, not surprising that the cichlid species flocks from the East African Great Lakes have received considerable attention as model systems for the study of adaptive radiation and explosive speciation [6,13,14].

The family Cichlidae is, however, just one of several families of freshwater fishes that inhabit the East African Great Lakes [1,15]. In Lake Tanganyika, the oldest lake in East Africa, more than 100 non-cichlid fish species in at least twenty families are recognized, about half of the species being endemic [1,16,17] (Table 1). Interestingly, all Tanganyikan fish families are known to also occur in the Congo River basin that is definitely older and holds a much larger number of fish families than any of the East African water-bodies [15,18]. An initially West African to Central African origin has therefore been proposed for the East African representatives of many of these families [18]. So far, such a scenario has only been confirmed with phylogenetic analyses for the Cichlidae [9,12,19-22], and Clupeidae (Wilson & Meyer, unpublished), while the Clariidae seem to have colonized Africa from the Arabian plate [23]. Eleven non-cichlid families with about 50 species have been described for Lake Malawi. Only one of these families is not represented in lakes Tanganyika and Victoria: A single anguillid species, the African mottled eel (*Anguilla bengalensis labiata*), migrates to the area of Lake Malawi via the Zambezi and Shire River. In Lake Victoria, representatives of thirteen non-cichlid fish families are known, two of which were introduced only recently. All other families are also found in Lake Tanganyika (Table 1). Aside of the Cichlidae, nine other families are known to live in all three Great Lakes: Bagridae, Characidae, Clariidae, Cyprinidae, Cyprinodontidae, Mastacembalidae, Mochokidae, Mormyridae, and Protopteridae.

**Table 1:** Freshwater fish families in the East African Great Lakes Victoria, Malawi and Tanganyika (according to refs. [1,16,17] and )

**fish family**	**Lake Victoria**	**Lake Malawi**	**Lake Tanganyika**
Amphiliidae		√	√
Anabantidae	√		√
Anguillidae		√	
Bagridae	√	√	√
Centrarchidae	√*		
Centropomidae	√*		√
Characidae	√	√	√
Cichlidae	√	√	√
Citharinidae			√
Clariidae	√	√	√
Clupeidae			√
Cyprinidae	√	√	√
Cyprinodontidae	√	√	√
Kneriidae			√
Malapteruridae			√
Mastacembalidae	√	√	√
**Mochokidae**	√	√	√
Mormyridae	√	√	√
Polypteridae			√
Protopteridae	√	√	√
Schilbeidae	√		√
Tetraodontidae			√

*introduced

We were interested in the phylogeny and phylogeography of one genus of the catfish family Mochokidae, the squeaker catfishes (*Synodontis*), in East Africa. With about 190 species assigned to ten genera, the siluriform family Mochokidae is endemic to the African continent where these fishes inhabit freshwaters from small creeks to large streams and from small ponds to large lakes. The genus *Synodontis *Cuvier, 1816 (including the monotypic genera *Brachysynodontis *and *Hemisynodontis*) comprises about 120 species, and is the most species-rich and widespread genus of the Mochokidae. *Synodontis *species are found throughout Africa, except in the southernmost parts and the Maghreb, although most species occur in Central and West Africa [24]. They are called "squeakers" (also by many local tribes), because of the noise they produce when taken out of the water. Some larger *Synodontis *species are important food resources. Other species are traded as ornamental fish due to their coloration and because of the interesting behavior of some of the species. The upside-down catfishes (*S. contractus*, *S. nigriventris*), for example, show an inverse swimming posture (Fig. 1b). Even more bizarre is probably the brood parasitic cuckoo catfish from Lake Tanganyika (*S. multipunctatus*) that cedes its eggs among those of spawning mouthbrooding cichlids; the female cichlid inadvertently takes up the catfish-eggs into her buccal cavity, together with her own eggs, where the young catfishes nourish on the cichlid larvae (Fig. 1c) [25].

Our interest in the East African *Synodontis *was founded on several reasons: Firstly, the Mochokidae are abundant in all three East African Great Lakes and are, as a consequence, an ideal system for comparative phylogenetic and phylogeographic analyses with the existing data on cichlid fishes. Secondly, of the nine non-cichlid families common to the three East African Great Lakes, the Mochokidae show the closest overlap in habitat in comparison to cichlids, in that they occur primarily in the littoral and sub-littoral zone [1,16]. Thirdly, among the two East African mochokid genera, *Synodontis *is more species rich as compared to *Chiloglanis*, particularly in Lake Tanganyika, where seven species of *Synodontis *have been described. Finally, most *Synodontis *species can be considered as euryphagous, which enables them to cope with seasonal changes in food abundance and habitat change [26,27]. Their feeding regime broadens their food niche and gives them a better ability to colonize different habitats as compared to more specialized fish species such as many lake-adapted cichlids. Thus, their colonization routes in East Africa should be good indicators of fish-accessible waterways in that region over evolutionary time spans.

In order to obtain a better understanding of the evolution, diversity and phylogeography of East African squeaker catfish, we aimed to reconstruct the phylogeny of East African *Synodontis *with special emphasis on the sequence and timing of colonization events of the East African Great Lakes Tanganyika, Malawi and Victoria, and the evolution of "cuckooing" behavior in Lake Tanganyika. Therefore, we sequenced a portion of about 900 bp of the mitochondrial genome of 21 species of the genus *Synodontis *and the outgroup taxa *Microsynodontis batesii *and *Chiloglanis sp*. We included all but one described, plus one, until now, undescribed, species from Lake Tanganyika, the single described Lake Malawi *Synodontis*, one of the two species occurring in Lake Victoria, two species from East and South African river systems, as well as eleven representatives of Central and West African species (Table 2).

**Table 2:** List of specimens used for this study

**Taxon**	origin*	**sample locality**	collector**	**Taxon ID**	**GenBank acc. nr. CR/ND6**	
*Synodontis njassae*	EA (LM)	M'bamba Bay	EV	MbBay (1995)	DQ663012	DQ663077
			EV	Mst45 (1995)	DQ663023	-
			EV	M56M5 (1995)	DQ663007	DQ663070
			EV	M5336 (1995)	DQ663005	DQ663068
			EV	M5350 (1995)	DQ663006	DQ663069
			EV	M7043 (1995)	DQ663008	DQ663071
			EV	M9008 (1995)	DQ663009	DQ663072
			EV	M9016 (1995)	DQ663022	-
			EV	M9043 (1995)	DQ663010	DQ663073
			EV	M9076 (1995)	-	DQ663074
			EV	M9092 (1995)	DQ663011	DQ663075
			EV	M9096 (1995)	DQ663024	-
			EV	T995 (1995)	DQ663025	-
*Synodontis victoriae*	EA (LV, LK, M)	Kisumu, LV	WS	5i8a (2004)	DQ663013	DQ663059
		Kisumu, LV	WS	5i8b (2004)	DQ663014	DQ663060
*Synodontis dhonti*	EA (LT)	Tanganyika Lodge	SK, CS	3698	DQ662994	-
		Kasakalawe Lodge	SK, CS	3708	DQ662995	-
		Kasakalawe Lodge	SK, CS	3709	DQ662996	-
		Chituta Bay	SK, CS	3736	DQ662997	DQ663056
		Mtondwe Island	SK, CS	3738	DQ662998	-
		Katoto	SK, CS	3771	DQ662999	-
		Kasakalawe	SK, CS	3777	DQ663000	DQ663057
		Kasakalawe	SK, CS	3778	DQ663001	-
		Kalambo Lodge	SK, CS	4535	DQ663002	-
		Chituta Bay	TV	4536	DQ663003	-
		Chituta Bay	TV	4537	DQ663004	-
		Ulwile Island	EV	TB 127	DQ663019	DQ663065
		Ulwile Island	EV	TB 128	DQ663020	DQ663066
*Synodontis granulosus*	EA (LT)	Chituta Bay	SK, CS	3735	DQ662961	-
		Mtondwe Island	SK, CS	3773	DQ662962	DQ663053
*Synodontis multipunctatus*	EA (LT)	Muzumwa	SK, CS	3635	DQ662963	DQ663046
		Kalambo Lodge	SK, CS	3696	DQ662964	-
		south of Kalambo	SK, CS	3697	DQ662965	-
		Mpulungu	SK, CS	3712	DQ662968	-
		Mpulungu	SK, CS	3714	DQ662969	-
		Mpulungu	SK, CS	3715	DQ662966	-
		Mpulungu	SK, CS	3716	DQ662967	-
		Kasakalawe	SK, CS	3737	DQ662970	-
		?***	SK, CS	3739	DQ662971	DQ664047
		Muzumwa	SK, CS	4112	DQ662972	-
		Muzumwa	SK, CS	4113	DQ662973	-
		Muzumwa	SK, CS	4114	DQ662974	-
		Chituta Bay	TV	4538	DQ662975	-
		Ulwile Island	EV	TB 073	-	DQ663062
		Ulwile Island	EV	TB 125	DQ663015	DQ663063
		Ulwile Island	EV	TB 126	DQ663016	DQ663064
		south of Mkangansi	EV	TB 872	DQ663017	DQ663067
*Synodontis petricola*	EA (LT)	Kapembwa	SK, CS	3636	DQ662976	DQ663048
		Chituta Bay	SK, CS	3699	DQ662977	DQ663050
		Mpulungu	SK, CS	3731	DQ662978	-
		Mpulungu	SK, CS	3732	DQ662979	-
		Katoto	SK, CS	3769	DQ662980	-
		Katoto	SK, CS	3770	DQ662981	-
*Synodontis polli*	EA (LT)	Kalambo Lodge	SK, CS	3695	DQ662983	DQ663049
		Kasakalawe Lodge	SK, CS	3710	DQ662984	-
		Kasakalawe Lodge	SK, CS	3711	DQ662985	-
		Mpulungu	SK, CS	3717	DQ662986	-
		Mpulungu	SK, CS	3733	DQ662987	DQ663052
		?***	SK, CS	4030	DQ662988	-
		Mtondwe Island	SK	4539	DQ662982	-
		Ulwile Island	EV	TB 072	DQ663018	DQ663061
*Synodontis sp. nov.*	EA (LT)	Mpulungu	SK, CS	3718	DQ662989	DQ663051
		Mpulungu	SK, CS	3775	DQ662990	DQ663054
		Gombe	SK, CS	3776	DQ662991	-
		?***	SK, CS	4031	DQ662992	DQ663055
		Tanganyika Lodge	SK, CS	4532	DQ662993	-
*Synodontis nigromaculatus*	EA (LT, R)	Lake Mweru	RS	4342	DQ662954	DQ663043
		Lake Mweru	RS	4339	DQ662955	DQ663045
*Synodontis zambezensis*	EA (R)	Cinzombo Lodge; Luagwa R.	CK	3977	DQ662952	-
		Nsefu Lagoon; Luangwa R.	CK	3978	DQ662953	DQ663039
*Synodontis angelicus*	CA (Congo R.)	?***	SK	3744	DQ662944	DQ663033
		?***	SK	3745	DQ662945	-
*Synodontis brichardi*	CA (Congo R.)	?***	SK	3993	DQ662943	DQ663035
*Synodontis eupterus*	WA, Nile	?***	SK	3861	DQ662956	DQ663036
		?***	SK	4088	DQ662957	-
		?***	SK	4108	DQ662958	DQ663037
*Synodontis flavitaeniatus*	CA (Congo R.)	?***	JS	75	-	DQ663077
*Synodontis nigrita*	WA, Nile	?***	SK	3868	DQ662947	DQ663038
		?***	SK	4337	DQ662948	DQ663041
		?***	SK	4338	DQ662949	DQ663042
*Synodontis nigriventris*	CA (Congo R.)	?***	SK	3700	DQ662941	DQ663030
		?***	SK	3701	DQ662942	-
		?***	JS	56	DQ663021	DQ663058
*Synodontis ocellifer*	WA	?***	SK	3994	DQ662960	DQ663031
*Synodontis polystigma*	CA (Congo R.)	Lake Mweru	RS	4341	DQ662951	DQ663044
*Synodontis robbianus*	WA (Niger R.)	?***	SK	3702	DQ662938	DQ663029
		?***	SK	3703	DQ662939	-
		?***	SK	4089	DQ662940	-
*Synodontis robertsi*	CA (Congo R.)	?***	SK	4336	DQ662946	DQ663040
*Synodontis schoutedeni*	CA (Congo R.)	?***	SK	3860	DQ662950	DQ663034
*Synodontis velifer*	WA	?***	WK	3995	DQ662959	DQ663032
*Chiloglanis sp.*		Lunzua R.	SK, CS	3865	DQ662935	DQ663026
*Microsynodontis batesii*		?***	SK	4024	DQ662936	DQ663027
		?***	SK	4025	DQ662937	DQ663028

*CA, Central Africa; LK, Lake Kivu; LM, Lake Malawi; LT, Lake Tanganyika; LV, Lake Victoria; M, Malagarazi River; R, East African rivers; WA, West Africa;

**CS, Christian Sturmbauer; CK, Cyprian Katongo; EV, Erik Verheyen; JS, Jos Snoeks; RS, Robert Sinyinza; SK, Stephan Koblmüller; TV, Toby Veall; WK, Walter Köhldorfer; WS, Walter Salzburger

***samples obtained through ornamental fish trade.

Coordinates of sampling localities (when available):

LT – Chituta Bay, S 08°44' E 31°09'; Gombe, S 08°47' E 31°01'; Kalambo Lodge, S 08°39' E 31°13'; Kapembwa, S 08°37' E 30°51'; Kasakalawe, S 08°47' E 31°04'; Kasakalawe Lodge, S 08°47' E 31°06'; Katoto, S 08°48' E 31°01'; Mpulungu, S 08°46' E 31°06'; Mtondwe Island, S 08°43' E 31°07'; Muzumwa, S 08°42' E 31°12'; south of Kalambo, S 08°37' E 31°12'; south of Mkangansi, S 06°37' E 30°18'; Tanganyika Lodge, S 08°47' E 31°05'; Ulwile Island, S 07°72' E 30°34'Lunzua R. (near Mwanangwa), S 08°57' E 31°10'

## Results

**Figure 2:**
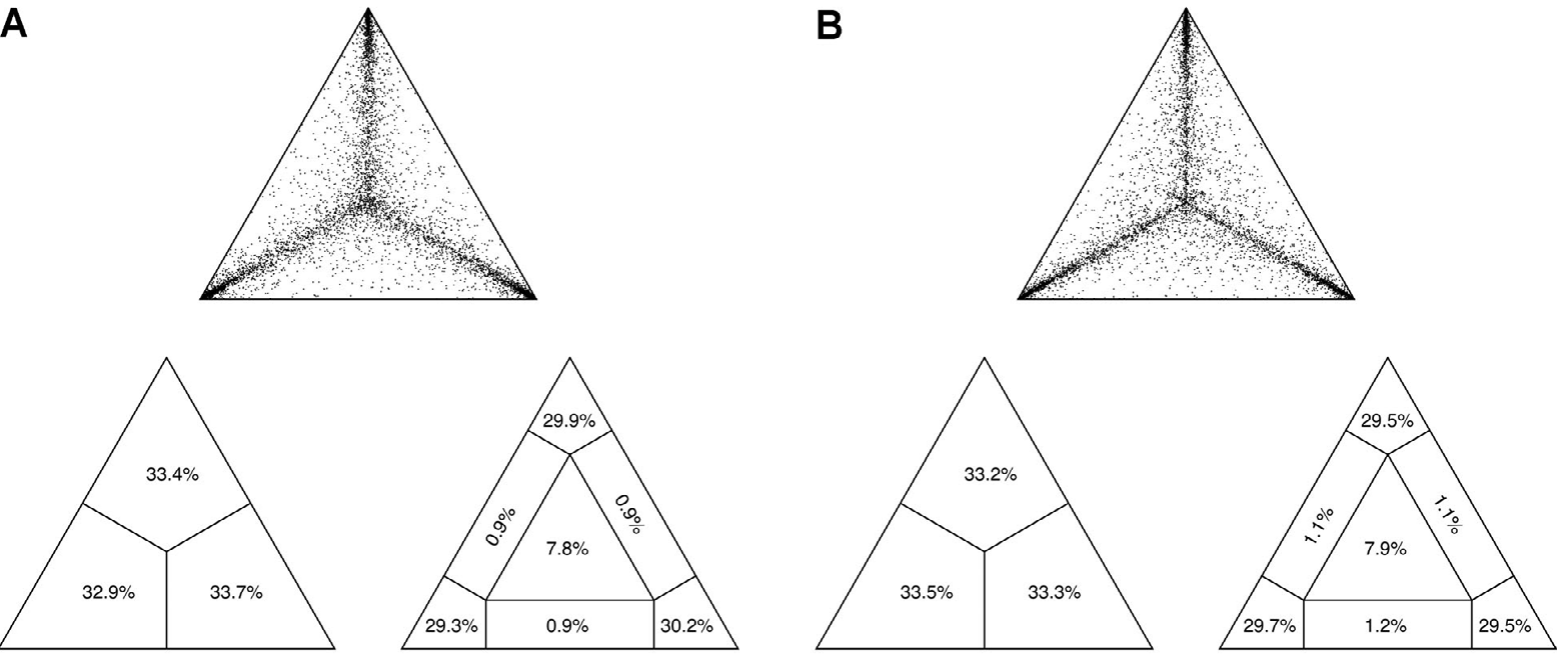
**Results from the likelihood mapping analysis**. (*a*) In theanalysis of the entire dataset, a percentage fraction of 89.4 % of all quartets was fully resolved (and 7.8 % were not resolved). (*b*) In the reduced dataset, 88.7 % of the quartets were resolved (7.9 % were not resolved). This points to a strong phylogenetic signal in the data.

For this study, we analyzed 407 bp of the mitochondrial control region of 94 specimens and 426 bp of the NADH dehydrogenase subunit 6 gene (ND6) of 52 specimens (including the outgroup taxa). The likelihood mapping analysis yielded a fraction of 89.4% fully resolved quartets for the total combined data set (Fig. 2a), and 88.7% fully resolved quartets for the subset including the East African taxa only (Fig. 2b), indicating a fairly strong phylogenetic signal in both data sets. Within the genus *Synodontis*, pairwise distances (uncorrected p-distances) ranged up to 33.3% (*S. shoutedeni versus S. multipunctatus*) in the control region and up to 11.5 % (*S. granulosus versus S. robertsi*) in the ND6 gene segment. The largest uncorrected p-distances within the East African clade were 23.7% in the control region and 5.9% in ND6 (in both cases involving the species pair *S. njassae *and *S. multipunctatus*). The two gene segments analyzed, thus, corroborate the observation that in the mitochondrial genome the non-coding control region generally evolves about two to five times faster than protein coding genes [28].

**Figure 3:**
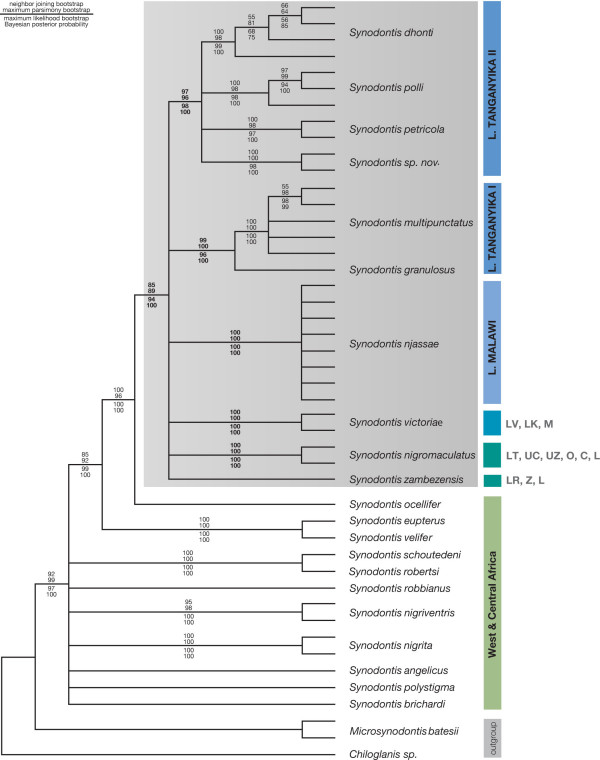
**Composite consensus tree of the phylogenetic analyses**. The strictconsensus of the neighbor-joining tree, the most parsimonious trees, the optimal maximum likelihood topology (see Fig.4) and the Bayesian inference tree is shown. Numbers above the branches are neighbor-joining and maximum parsimony bootstrap values, numbers below the branches represent maximum likelihood bootstraps and Bayesian posterior probabilities. The grey box indicates the East African clade of *Synodontis*.

**Figure 4:**
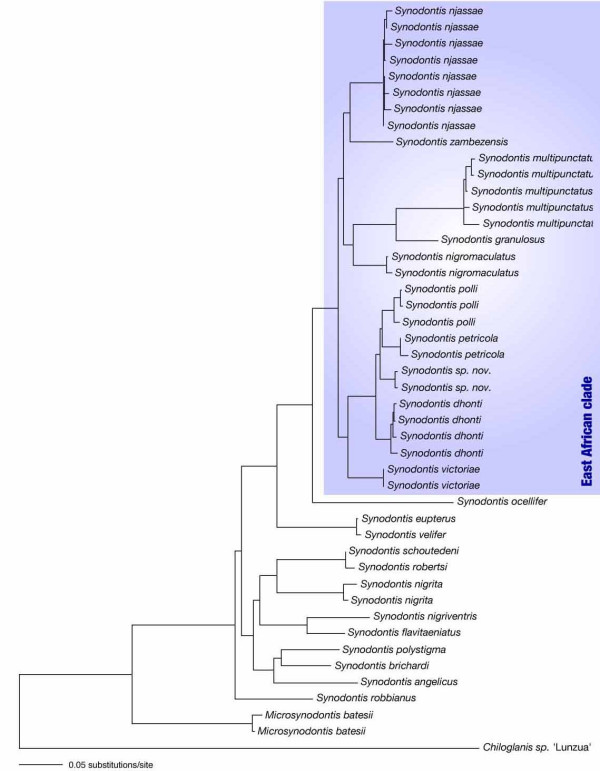
**Maximum likelihood tree**. Maximum likelihood topology based on the K81uf+I+Γ model of molecular evolution [70] with nucleotide frequencies A, 0.3581, C, 0.2676, G, 0.1368, T, 0.2375, proportion of invariable sites (I), 0.2461, gamma shape parameter (α), 0.7306, and R-matrix A↔G, A↔T, C↔G and G↔T, 1.0000; A↔G, 7.7875 and C↔T, 1.2463. The blue box indicates the East African clade of *Synodontis*.

In our phylogenetic analyses, the internal branches interrelating major Central and West African lineages were short and, hence, the tree topologies remained somewhat unresolved and the branching topology varied depending on the tree-building algorithm used. This seems to suggest that several Central and West African lineages originated almost contemporaneously in the course of a major cladogenetic event. However, a much more thorough sampling and the analysis of a more slowly evolving gene segment (*e.g.*, RAG1 [29]) would be necessary to shed light on this part of the evolutionary history of *Synodontis *in Africa. Our analyses consistently revealed that the Central and West African members of the genus *Synodontis *are ancestral to the East African representatives. The monophyly of the East African clade is clearly seen in the composite consensus tree (Fig. 3) based upon six most parsimonious trees [tree length, 49,875 steps; consistency index (CI) excluding uninformative characters, 0.47; retention index (RI), 0.74; rescaled consistency index (RC), 0.38], the neighbor joining (NJ) tree, the maximum likelihood (ML; Fig. 4) tree and the Bayesian inference phylogeny. The monophyly of the East African representatives was further confirmed by the individual analysis of the mitochondrial gene segments, the control region and the ND6 gene (trees not shown).

**Figure 5:**
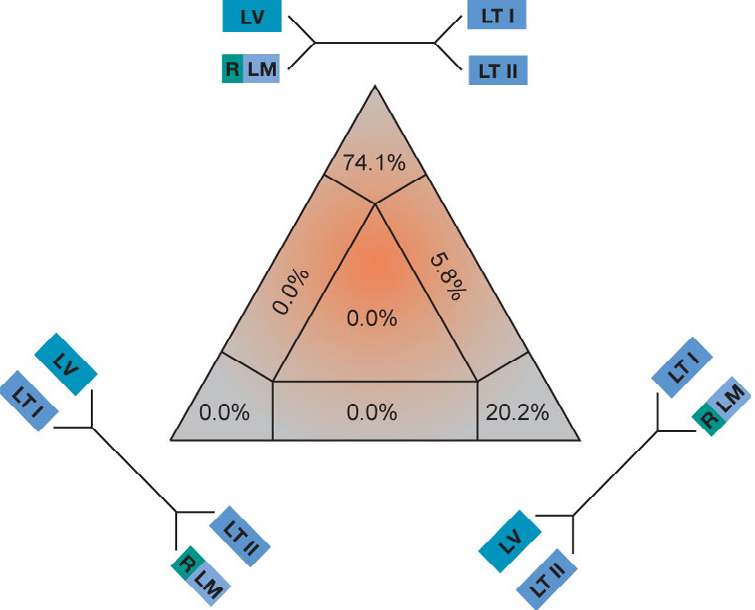
**Four-cluster likelihood mapping analysis**. The topology with a monophyly of the two endemic Tanganyikan clades receives the highest support. LM, Lake Malawi (*S. njassae*); LT I, first Lake Tanganyika clade; LT II, second Lake Tanganyika clade; LV, Lake Victoria (*S. victoriae*); R, riverine representatives.

The phylogenetic analyses of the East African *Synodontis *species revealed a similar outcome compared to that of the ancestral Central and West African lineages, with relatively short branches interrelating the main lineages (and low bootstrap support and posterior probabilities for these branches), indicating an almost contemporaneous origin of the main East African *Synodontis *lineages. Six distinct mitochondrial lineages can be distinguished within the East African clade of *Synodontis*. The species *S. nigromaculatus*, *S. zambezensis*, *S. njassae *and *S. victoriae *constitute separate clades, while the remaining Lake Tanganyika species can be divided into two distinct genetic lineages. The first Tanganyikan clade contains *S. granulosus *and the cuckoo catfish (*S. multipunctatus*); the second Tanganyikan clade includes *S. dhonti*, *S. petricola*, *S. polli *and *S. sp. nov*. In the maximum parsimony (MP) and NJ tree of the reduced dataset, the two clades of Lake Tanganyika *Synodontis *were resolved as a monophyletic group, albeit with low bootstrap support. In ML and BI, on the other hand, the two Tanganyikan clades were not resolved as sister groups, and *S. victoriae *was placed as sister group to the clade comprising *S. dhonti*, *S. petricola*, *S. polli *and *S. sp. nov. *– with similarly low support values. The four-cluster-likelihood-mapping (Fig. 5) favors a topology with a monophyletic origin of Lake Tanganyika *Synodontis*, and *S. victoriae *being more closely related to *S. nigromaculatus*, *S. zambezensis *and *S. njassae*. A Shimodaira-Hasegawa test [30] based on the tree topologies obtained from the reduced data set revealed that there are no significant differences between the topologies obtained by the different algorithms (P < 0.05).

Within the second Tanganyikan clade the branching order among the four species *S. dhonti*, *S. petricola*, *S. polli*, *S. sp. nov. *differed with respect to the tree-building algorithm used. Together with the low bootstrap values and posterior probabilities this again points to an event of rapid cladogenesis [31]. In the other Tanganyikan clade, we observed relatively long branches (see Fig. 4), and also the uncorrected pairwise distances indicated a faster evolutionary rate in the cuckoo catfish (see above). For example, the maximum uncorrected pairwise distance in the control region in *S. multipunctatus *was 6.5%, whereas in *S. njassae *a maximum uncorrected pairwise distance of only 1.5% was found. Relative rate tests with RRTree indeed recovered a significantly higher (P < 0.01) substitution rate for *S. multipunctatus *compared to all other East African species. This was found for both gene segments.

In order to tentatively date the major cladogenetic events in *Synodontis *in East Africa, we used r8s [32] and applied different calibration points for our analyses. In our first round of analysis, in which we applied all available dates, we obtained an age of 12.24 million years (MY) for the most recent common ancestor (MRCA) of *S. granulosus *and *S. multipunctatus *(minimum estimate: 11.29 MY; maximum estimate: 13.17 MY), and an age of 2.15 MY (min.: 0.97; max.: 2.37) for the MRCA of the cuckoo catfish (*S. multipunctatus*) (see Table 3). When applying two calibration points only, we obtained highly similar values plus an age of 7.5 MY for the second Tanganyikan clade (min.: 5.48; max.: 12.11). The MRCA estimates did not change substantially when only the fossil calibration was used (Table 3). The age for the entire genus was estimated with ca. 35 MY (min.: 22.87; max.: 40.12). This result should, however, be interpreted with caution since these dates lie outside the range of our calibration points, and since we might not have represented the entire diversity of *Synodontis *in Central, South and West Africa.

**Table 3:** Dating of the major cladogenetic events in *Synodontis *with r8s. We ran three independent analyses in r8s [32] with different calibration points using the maximum estimated age for the lacustrine habitat in lakes Malawi (1 MY [49]) and Tanganyika (6 MY [46–48]), as well as the minimum age of the East African clade of *Synodontis *as suggested by the oldest known *Synodontis *fossil in that region (>20 MY [45]). In the first analysis, all three calibrations were applied (1/6/20 calibration); in the second cycle, we used the Lake Malawi and the fossil calibration (1/20 calibration); in the third round, we only used the fossil based calibration (20 calibration). The numbers indicate the average value (in MY) obtained from a bootstrap approach with 30 replicates, the minimum and maximum values are depicted in round brackets (in italics). Square brackets indicate the time constraints used for the different r8s analyses and the range of the actual numbers used in the bootstrap replicates (in round brackets). The estimates for the age of the entire genus *Synodontis *should be interpreted with caution, as the values lie outside our range of calibration points

**Clade**	**1/6/20 MY calibration**	**1/20 MY calibration**	**20 MY calibration**
*Synodontis*	34.54 (*23.42–38.14*)	35.39 (*24.01–38.53*)	37.26 (*22.87–40.12*)
East African clade	[>20 (20–20.49)]	[>20 (20–20.45)]	[>20 (20–22.53)]
L. Tanganyika I	12.24 (*11.29–13.17*)	12.36 (*11.36–13.29*)	12.89 (*11.24–14.81*)
L. Tanganyika II	[<6 (4.77–6)]	7.5 (*5.48–12.11*)	7.88 (*5.76–12.36*)
S. multipunctatus	2.15 (*0.97–2.37*)	2.21 (*1.61–2.43*)	2.31 (*1.64–2.58*)
L. Malawi (*S. njassae*)	[<1 (0.99–1)]	[<1 (0.99–1)]	1.75 (*1.34–2.18*)

## Discussion

### Mitochondrial phylogeny of the East African *Synodontis *species

Although the genus *Synodontis *is the most species-rich and widespread genus of the Mochokidae accounting for about a quarter of all African siluriform species, there is no molecular phylogeny available for this group. Earlier attempts based on a small number of sequences of the mitochondrial cytochrome *b *gene revealed a sister-group relationship between an East African clade (represented by *S. zambezensis, S. nigromaculatus, S. njassae*, and *S. petricola*) and some South-African representatives [33]. However, no Central and/or West African taxa have been included in this analysis that would allow placing the investigated species into a larger phylogenetic and phylogeographic context.

We were interested in the phylogeny and phylogeography of the East African *Synodontis *species and, therefore, combined the mtDNA sequences of East African representatives with those of eleven Central and/or West African species. Our analyses of the combined dataset as well as the separate analyses of each mitochondrial locus, consistently revealed the monophyly of the East African clade and its derived position compared to the Central and/or West African taxa (Figs. 3, 4). While the phylogenetic relationships between most of the more ancestral Central and/or West African representatives could not be resolved, which is most likely due to the large genetic distances among these taxa, we could identify a sister-group relationship between a clade comprised by *S. velifer *and *S. eupterus *and *S. ocellifer *plus the East African clade (Figs. 3, 4).

Within the East African clade, we could clearly identify six distinct lineages: *S. zambezensis*; *S. nigromaculatus*; *S. victoriae *(Lake Victoria); *S. njassae *(Lake Malawi); *S. granulosus *and *S. multipunctatus *(Lake Tanganyika I); *S. dhonti, S. polli, S. petricola, S. sp. nov. *(Lake Tanganyika II). However, it was not possible to resolve the phylogenetic relationships between these lineages, as indicated by the short branches interconnecting them, the lacking statistical support for these branches, and the fact that different tree building algorithms led to different phylogenetic hypotheses with regard to the relative position of these lineages. Such a lack of phylogenetic signal in ancestral branches is frequently observed in cases of relatively rapid lineage splitting events [12,22,29,31,34-36], *e.g.*, as consequence of the synchronized fragmentation of a founder population into allopatric units [31]. We, thus, suggest a scenario for the evolution of *Synodontis *in East Africa, in which one ancestral (colonizing) species was split into at least six sub-lineages within a relatively short period of time, and that – at least in some cases – these sub-lineages diversified further, albeit in isolation from each other. In this context, it is interesting to note that hybrids can artificially be produced between two of the lineages, the riverine taxa *S. zambezensis *and *S. nigromaculatus *[33,37]. It is, however, unlikely that hybridization distorted our phylogenetic signal, as most of the lineages exclude each other geographically so that no natural hybridization was and is possible. The specimens of the only two species that partially overlap in range (*S. zambezensis *and *S. nigromaculatus*) showed reciprocal monophyly and appeared as distinct evolutionary units with an average sequence divergence of ca. 12% in the control region and ca. 4.5% in ND6.

Lake Tanganyika is the only East African lake that harbors a small assemblage of endemic *Synodontis *species [16,17]. Our phylogenetic analyses indicate the existence of two separate lineages of *Synodontis *in Lake Tanganyika (see Fig. 3) (note that also the non-endemic *S. nigromaculatus *is found in that lake). The first lineage comprises the relatively large Tanganyikan representative *S. granulosus *and the cuckoo catfish (*S. multipunctatus*), a brood parasite of mouth-brooding cichlids [25] (Fig. 1c). The second Tanganyikan clade includes the species *S. dhonti, S. polli, S. petricola*, and a yet undescribed species. There are some indications, *e.g., *the four-cluster likelihood mapping analysis (Fig. 5), for a monophyly of the two Lake Tanganyikan clades, although statistical support in the individual bootstrap analysis and in the Shimodaira-Hasegawa test is lacking. While in both Tanganyikan clades the monophyly of the taxa was supported with high bootstrap values and Bayesian posterior probabilities, the phylogenetic relationships between the four lineages of the second Tanganyikan clade could not be resolved with high confidence (Fig. 3). We interpret these short branches as the result of the relatively rapid lineage splitting at the beginning of the formation of Lake Tanganyika. It also seems possible that some hybridization took place in the early phases of species formation (see *e.g.*, [38]), which could be tested with nuclear DNA sequences. However, it is unlikely that the observed lack of resolution in some branches interrelating main lineages (or taxa) is due to the markers used, since, overall, we observe a sufficiently strong phylogenetic signal in the dataset (Fig. 2), and since the control region is the fastest evolving section of the mitochondrial genome.

### Phylo-chronology of *Synodontis *in East Africa

The reconstruction of divergence times from molecular data or phylogenies is not without potential pitfalls (see *e.g., *[39,40]). Yet, such time estimates are useful for providing an approximate framework that puts diversification events into a temporal framework. Our phylo-chronological reconstruction of the major cladogenetic events in the East African clade of *Synodontis *(Table 3) is based on three independent calibration points, which were used in different combinations. Because these calibration points are derived from estimates, it is important to note that our approximations should be interpreted cautiously. For example, we used a minimum age of 20 MY for the MRCA of the East African clade, based on the oldest known fossils in that area [41-45]. These Early Miocene fossils are, however, not particularly well dated (although 20 MY seems like a good approximation [43]), they have not been assigned to extant species, and the possibility always remains that still older fossils exist. However, together with the internal calibration pointes – the ages of lake basins – the estimated scenario for the evolution and phylogeography of *Synodontis *in East Africa is congruent with geological and paleolimnological events.

With regard to the entire East African clade of *Synodontis*, the oldest known fossils from the Early Miocene are relatively close to the estimated age of the MRCA of that clade. In our bootstrap replicates, the age of the MRCA of the East African clade was estimated between 20 (the constrained minimum age in the analyses) and 22.53 MY. For the MRCA of the first Tanganyikan clade, with *S. granulosus *and *S. multipunctatus*, we obtained an age of 11.24–14.81. This is older than the age of the onset of a truly lacustrine deepwater habitat in Lake Tanganyika [46-48], suggesting that the split between these two taxa took place before the ecosystem of a deep tropical lake has formed (*e.g.*, in the riverine environment of the Congo and/or Proto-Malagarazi River) or, possibly more likely, a colonization in the initial period of lake formation when extensive swampy areas with shallow basins are likely to have existed in the area of present Lake Tanganyika. Thus, although both *S. granulosus *and, particularly, the cuckoo catfish (*S. multipunctatus*) are highly specialized lake species, their ancestors were riverine fish that seem to have independently achieved their lacustrine lifestyle. In contrast, the second Tanganyikan clade appears to have undergone its primary radiation at a later stage, most likely in a lacustrine (or semi-lacustrine) environment (ca. 5–8 MYA; although the maximum values from individual bootstrap replicates indicate that even older estimates are possible). The only independent estimation for the MRCA of *S. njassae *from Lake Malawi revealed an age of 1.34–2.18 MY. We find that the cuckoo catfish (*S. multipunctatus*) has diverged after about 2 MYA (0.97–2.58 MYA). Interestingly, this would be immediately after the host lineage of the cuckoo catfish, the mouthbrooding haplochromine cichlids, has initiated its radiation in Lake Tanganyika, according to a recent calibration [9].

Our strategy of carrying out the analysis in three cycles also aimed to test the validity of some calibration points, such as the age of the MRCA within Lake Malawi and the second clade in Lake Tanganyika. While our analyses revealed highly consistent results for the MRCA of the first Tanganyikan clade and that of *S. multipunctatus *(Table 3), the estimates for the MRCA of *S. njassae *(Lake Malawi) and that of the second Tanganyikan clade are slightly older than expected based on the presumed formation of the truly lacustrine habitat in both lakes [46-49]. This could, of course, be due to analytical limitations in estimating divergence times based on molecular phylogenies. Alternatively, it may indicate the actual coalescence age of the riverine MRCA of both lineages. However, it could also mean that the lake basins are somewhat older or that the divergence of *Synodontis *in the basins of lakes Malawi and Tanganyika began before the establishment of the present lacustrine habitat with its characteristic deep-water conditions, *e.g., *in more shallow pools in the transition phase between a swampy habitat of a proto-lake and a real lake.

### Phylogeography of *Synodontis *in East Africa

Based on our phylogenetic and phylo-chronological analysis of *Synodontis *in East Africa, we can outline the following overall phylogeographic scenario: It seems that after an initial colonization of East Africa through a potentially widespread Central and/or West African representative (probably about 20 MYA), at least six lineages of *Synodontis *evolved that are each confined to particular geographic regions. The rapid spread of East African *Synodontis *can be explained by a colonization of the area via the ancient Congo-Malagarazi River connection, and incidents of drainage catchments towards the Nile systems (in the North) and between the Upper Congo and Zambezi drainage (in the South) (see ref. [50] and citations therein). For example, the two riverine species *S. nigromaculatus *and *S. zambezensis *are widely distributed in Southern and Eastern Africa (but are phylogenetically distinct from the South African clade [33]). The black-spotted squeaker catfish (*S. nigromaculatus*) is found in the Cunene River, the Okavango River, the upper reaches of the Congo River, the upper Zambezi basin, the Limpopo as well as in Lake Tanganyika, demonstrating the existence of fish accessible water-ways through-out eastern and southern Africa until recently (see *e.g.*, [9,35]). The plain squeaker (*S. zambezensis*) is found in the Zambezi and Limpopo system, as well as in Lake Rukwa. The maximum-likelihood analysis (Fig. 4) suggested a sister-group relationship between the easternmost species, *S. zambezensis*, and *S. njassae *from Lake Malawi, and between *S. nigromaculatus *and the first Tanganyikan clade. Both hypotheses would, phylogeographically, make sense. However, a more detailed sampling and, particularly, much longer sequences would be necessary to confirm this hypothesis.

With our sampling strategy and phylogenetic analyses we are able to present a robust hypothesis for the evolution of *Synodontis *in East Africa (see above). However, we are not able to address questions related to the phylogeography of single species as has been undertaken for the clariid *Clarias gariepinus *[51]. It would, for example, be interesting to compare the phylogeography of *S. nigromaculatus *to one branch of haplochromine cichlids that have a comparable distribution range [9,35,50]. Similarly, a detailed phylogeographic scenario for *S. victoriae *and *S. zambezensis *could be used to test the existence of past fish-accessible connections between large East African lakes (see e.g., [9,10,18]). A much more thorough sampling of selected species and phylogeographic hypotheses for single species would be necessary in order to resolve these open questions.

### Comparative phylogeography between East African cichlids and squeaker catfishes

One of our goals for this study was to compare the phylogeny and phylogeography of the East African squeaker catfishes to the cichlid fishes, for which an impressive amount of data exists (see *e.g., *[9-12,35,52,53]). Our analyses reveal that, first of all, the initial diversification of *Synodontis *in East Africa is much older compared to the radiation of the extant cichlid species flocks in that area. (Note that there are representatives of more ancient cichlid lineages in East Africa; some of these have colonized East Africa secondarily [12,21].) In fact, the main lineages of *Synodontis *are likely to have existed before the oldest lake, Lake Tanganyika, has formed. A Central to West African origin is, however, likely for both groups, and it seems plausible that the ancestors of both the East African cichlid radiations and the East African *Synodontis *clade colonized the area at about the same time – with the Congo River system playing a zoogeographic key-role for the spread of both groups.

A main difference between the East African cichlids and *Synodontis *is also the evolutionary role that Lake Tanganyika has played. Lake Tanganyika is not only a reservoir for the most ancestral cichlid lineages in East Africa, but it has been identified as the cradle from which the most species-rich and widespread group of East African cichlids, the haplochromines, has evolved (see *e.g., *[9,12,54]). This "out of Tanganyika scenario" [9] implies that the species flocks of lakes Malawi and Victoria are derived from the older Tanganyikan radiation [9,12,54]. In *Synodontis*, we do not find evidence that a formerly endemic Tanganyikan lineage left the lake secondarily to colonize rivers and, subsequently, other lakes. Instead, Lakes Malawi and Victoria were seeded independently by ancestral lineages of *Synodontis*. Consequently, different and – in most cases – more ancient connections between water-systems were used by *Synodontis *to colonize East Africa.

There are, however, also similarities in the distribution of cichlids and squeaker catfishes. The range of the black-spotted squeaker catfish (*S. nigromaculatus*), for example, is similar to that of one lineage of haplochromine cichlids, the Congolese-South African clade (according to ref. [9]), and *S. zambezensis *overlaps with *Pseudocrenilabrus *[9,50]. A much more detailed sampling would be necessary to test for a putative congruence of time-scale and routes of colonization between these riverine cichlids and squeaker catfishes. Similarly, the occurrence of *S. victoriae *in lakes Kivu and Victoria seems to support the close relatedness of the fish faunas of both lakes [10]. A detailed phylogeographic scenario for *S. victoriae *would thus be of interest to further test for the commonality of geological and palaeolimnological events affecting several lineages of fishes.

### The evolution of brood parasitism in the cuckoo catfish (*S. multipunctatus*)

The most fascinating behavioral adaptation in *Synodontis *is certainly the evolution of brood parasitism in *S. multipunctatus *from Lake Tanganyika [25] (Fig. 1c). In the course of our sampling we found larvae of *S. multipunctatus *in the mouth of *Simochromis babaulti *and *S. diagramma *females (with up to three catfishes per female).

Our analyses resulted in two interesting findings regarding the evolution of cuckoo behavior in *S. multipunctatus*. First, our phylo-chronological analyses consistently revealed an age of about 2 MY for the MRCA of *S. multipunctatus. *This coincides with the MRCA of the mouthbrooding haplochromines of Lake Tanganyika [9], to which the current host-species of *S. multipunctatus *belong. It thus seems that brood parasitism evolved in *S. multipunctatus *at an early stage of the radiation of the Tropheini in Lake Tanganyika. Secondly, we find an accelerated rate of molecular evolution in both gene segments in the representatives of *S. multipunctatus*, as shown by longer branches in the phylogenies, larger genetic distances, and relative rate tests. A faster rate of DNA sequence evolution in brood parasites has been described in African finches, and it has been argued that this might be the consequence of a dynamic co-evolutionary history [55]. Iterative co-evolutionary processes between hosts and parasites might account for the observed significantly faster evolutionary rate in *S. multipunctatus*. Alternatively, there might be selective pressure to equalize the evolutionary rate in host-parasite systems. This could possibly explain why the mutation rate in the non-coding control region in *S. multipunctatus *approaches that observed in haplochromine cichlids [56] (6.5%; MRCA: 2 MYA), while the remaining representatives of the East African clade of *Synodontis *show considerably lower evolutionary rates (*e.g.*, *S. njassae*: 1.5%; MRCA: 1–1.5 MYA).

## Conclusion

Our phylogenetic and phylogeographic analyses of East African *Synodontis *species suggest the existence of at least six main lineages of *Synodontis *in East Africa that began to diversify about 20 MYA from a Central and/or West African ancestor. The six lineages show a clear-cut geographic pattern, and only the distributions of two riverine species, *S. nigromaculatus *and *S. zambezensis*, overlap. Representatives of three lineages are found in the oldest lake in the area, Lake Tanganyika, and two of these lineages are endemic and have undergone further diversification within the lake. The initial diversification of *Synodontis *in East Africa appears to be much older than that of the cichlid species flocks, yet, some taxa show similar geographic patterns compared to haplochromine cichlids, with which they often share the same habitat (in rivers and lakes). The endemic brood-parasitic cuckoo catfish (*S. multipunctatus*) from Lake Tanganyika seems to have diversified in parallel with its host species of the mouthbrooding modern haplochromines (the Tropheini). The significantly faster evolutionary rate in *S. multipunctatus *might be a consequence of co-evolutionary dynamics. Although *Synodontis *has colonized East Africa before the formation of the Great Lakes, which are home to the exceptionally diverse cichlid species flocks, and although the squeaker catfishes inhabit the same habitats as many of the cichlids, only a few species have evolved in one of the lakes. This, once more, illustrates the unparalleled, and still enigmatic, propensity of cichlid fishes to undergo explosive speciation.

## Methods

### Specimen information and DNA methods

This study is based upon a total of 91 individuals of 21 described and one so far undescribed species of *Synodontis*, mainly from Lakes Tanganyika, Malawi and Victoria, and several African river systems. As outgroup taxa we used one individual of *Chiloglanis sp. *and two individuals of *Microsynodontis batesii *[57,58]. Most of the specimens were sampled during several field expeditions from 1992 to 2004, while some additional samples were obtained from the aquarium trade (Table 2). Voucher specimens are available from the authors. Of all specimens, fin clips were taken and preserved in 96% ethanol.

We analyzed a 407 bp segment of the most variable part of the mitochondrial control region (D-loop) of 94 specimens (including the outgroup taxa) and 426 bp of the more slowly evolving NADH dehydrogenase subunit 6 gene (ND6) of 52 specimens (including the outgroup taxa). We chose the mitochondrial control region to be able to directly compare our present results to the East African cichlid species flocks, for which the control region is the most common mitochondrial DNA marker [9,10,12,35,50,59,60]. The ND6 gene was chosen because it has previously been used in clariid catfishes [51]. We decided not to use the mitochondrial cytochrome *b *gene, because previous analyses indicated a reduced phylogenetic signal in that gene with respect to the East African *Synodontis *species [33].

For DNA extraction we applied a proteinase *K *digestion followed by protein precipitation with ammonium acetate. As primers for amplification and sequencing of the most variable part of the mitochondrial control region we used L-Pro-F [61] and TDK-D [62]. For ND6 we used the primers ND5G and ND6L [63]. The PCR reactions were prepared for a total volume of 17 μl containing 0.085 μl of *Taq *DNA polymerase (BioTherm^TM^), 1.7 μl of each primer (10 μM), 1.7 μl 10x dNTP mix, 1.7 μl 10x buffer, 7.62 μl high performance liquid chromatography (HPLC) water, and 2.5 μl of the extracted DNA. Amplification was performed on a GeneAmp 9700 PCR system (Applied Biosystems) under the following conditions: an initial denaturation phase at 94°C for 3 min followed 45 cycles with denaturation at 94° for 30 sec, primer annealing at 50°C for 30 sec, and extension at 72°C for 1 min 30 sec, with a final extension phase at 72°C for 10 min. The PCR-products were purified with ExoSAP-IT (Exonuclease I and Shrimp Alkaline Phosphatase in buffer; Amersham Biosciences) prior to being added as template for chain termination sequencing following the protocol described in ref. [64]. DNA fragments were purified with Sephadex^TM ^G-50 (Amersham Biosciences) following the manufacturer's instruction and subsequently visualized on an ABI 3100 capillary sequencer (Applied Biosystems). All sequences are available from GenBank under the accession numbers listed in Table 2.

### Phylogenetic analysis

Alignment of the DNA sequences was performed using Clustal X [65] and improved by eye for the control region. Since all species were resolved as respectively monophyletic clades in a preliminary NJ tree (tree not shown) using all available sequences of the mitochondrial control region (calculated in PAUP* 4.0b10; [66]), we chose 52 taxa (including outgroup taxa) for amplification of the ND6 fragment. Several individuals had long insertions (> 100 bp) in the control region that were excluded for phylogenetic analyses. The combined dataset of control region and ND6, including 46 taxa, was used to reconstruct the phylogenetic relationships among the investigated *Synodontis *species. We applied neighbor-joining (NJ), maximum parsimony (MP) and maximum likelihood (ML) analyses in parallel, using the computer program PAUP*. Phylogenetic relationships were also reconstructed by Bayesian inference (BI) using MrBayes 3.1 [67].

To assess the degree of saturation of transition (ti) and transversion (tv) mutations at each codon position of the ND6 gene, we plotted the number of mutations against the percentage of sequence divergence of all pairwise comparisons (not shown). Based on the estimated ti/tv ratio inferred from these pairwise comparisons we derived a proper weighting scheme for a weighted MP analysis. Due to the estimated ti/tv ratio of 6.81 for third codon positions of two-and threefold degenerate amino acids, 2.63 for third codon positions of fourfold degenerate amino acids, 2.00 for second codon positions, and 1.75 for first codon positions we applied the following weighting scheme (tv/ti): 70:10 for third codon positions of two- and threefold degenerate amino acids, 26:10 for third codon positions of fourfold degenerate amino acids, 70:35 for second codon positions, and 70:28 at first codon positions. C/T substitutions at the first codon position of leucine were treated as a fifth base and were down-weighted to the same weight as transitions at the third codon positions. To achieve a proper weighting scheme for the control region, we first performed a sliding window analysis to define regions exhibiting different degrees of variation (<10%, low variable; 10–20%, high variable; >20%, hyper variable) [68]. Despite considerable saturation, transition mutations contain substantial information necessary to resolve the relationships of evolutionary young splits, without obscuring the deep splits when they are down-weighted according to their frequency in relation to transversion mutations [22]. We thus evaluated the degree of saturation of ti and tv for each partition of the control region separately, and based upon the estimated ti/tv ratios of 1.40, 2.43 and 2.28 for the low, high and hyper variable regions we obtained following weighting schemes (tv/ti): low variable, 70:56; high and hyper variable, 70:28. To evaluate an appropriate substitution model for the ML analysis, we calculated hierarchical likelihood ratio test statistics using the program Modeltest 3.06 [69]. The model recovered was K81uf+I+Γ [70] with nucleotide frequencies A, 0.3581, C, 0.2676, G, 0.1368, T, 0.2375, proportion of invariable sites (I), 0.2461, gamma shape parameter (α), 0.7306, and R-matrix A↔C=G↔T+1; A↔T+C↔G=1.2463; and A↔G=C↔T=7.7875. For obtaining MP and ML topologies we applied heuristic search procedures with random addition of taxa and TBR branch swapping (1,000 replicates for MP; 100 replicates for ML). As standard measures of confidence we applied bootstrapping (1,000 pseudo-replicates for NJ and MP, 100 for ML). For the reconstruction of phylogenetic relationships by Bayesian inference analyses our data were partitioned by gene and codon position (only in ND6). Rate heterogeneity was set according to a gamma distribution with six rate categories (GTR model) for each data partition. Posterior probabilities were obtained from a 2,000,000-generation Metropolis-coupled Markov chain Monte Carlo simulation (four chains; chain temperature, 0.2; trees sampled every 100 generations), with parameters estimated from each data partition. A 50% majority-rule consensus tree was constructed after a burn-in of 1%, which is when likelihood values reached stationarity.

To obtain a potentially better insight into the phylogenetic relationships among the East African member of the genus *Synodontis *we repeated the NJ, MP, ML and BI analyses with a reduced dataset including the species *S. dhonti*, *S. granulosus*, *S. multipunctatus*, *S. nigromaculatus*, *S. njassae*, *S. petricola*, *S. polli*, *S. sp. nov.*, *S. victoriae *and *S. zambezensis*, using *S. eupterus*, *S. velifer *and *S. ocellatus *as outgroup taxa, based on the first round of phylogenetic analyses. Proper weighting schemes for MP analysis were derived from estimated ti/tv-ratios as described above. Since no tv were observed for third codon positions of two-and threefold degenerate amino acids, we applied the same weighting scheme as in the overall data set for these codon positions. Based on the estimated ti/tv ratio of 2.49 for third codon positions of fourfold degenerate amino acids, 2.28 for second codon positions, and 1.19 for first codon positions we applied following weighting scheme (tv/ti): 25:10 for third codon positions of fourfold degenerate amino acids, 70:31 for second codon positions, and 70:59 at first codon positions. C/T substitutions at the first codon position of leucine were again treated as a fifth base and down-weighted to the same weight as transitions at the third codon positions. For the control region we obtained ti/tv ratios of 2.28, 3.20 and 2.18 for low, high and hyper variable regions resulting in a weighting (tv/ti) of 70:31, 70:22 and 70:32, respectively. For NJ, ML and BI, the best fit substitution model, recovered by the hierarchical likelihood ratio tests implemented in Modeltest 3.06 [69], was HKY+I+Γ [71] with base frequencies A, 0.3565; C, 0.2814; G, 0.1308; T, 0.2313; ti/tv, 4.7844; I, 0.3046; and α, 0.5463. A four-cluster likelihood mapping analysis [72] using the program TREE-PUZZLE 5.1 [73] was applied to test the hypothesis of the monophyly of the two endemic Tanganyikan clades.

To determine differences in substitution rates between East African representatives of the genus *Synodontis *and to test whether the cuckoo catfish (*S. multipunctatus*) indeed shows an accelerated rate of molecular evolution as indicated by its long branch, we conducted a relative rate test as implemented in RRTree [74]. We therefore tested *S. multipunctatus *against the remaining East African taxa, applying the K2P substitution model [75].

We used r8s [32] to tentatively date the major cladogenetic events in *Synodontis *in East Africa. We used the maximum likelihood trees and applied the local molecular clock method with an optimization via the truncated newton method [76]. The analyses were performed in three cycles with different calibration points, and 30 bootstrap replicates for each cycle (the files for this bootstrapping approach were generated with Mesquite [77]; the maximum likelihood trees were generated with PAUP* [66] as described above). In the first cycle, we used all three available calibration points: (*i*) the maximum age of about one million years for the lacustrine radiation of the Lake Malawi species *S. njassae*, based on the age of the truly lacustrine habitat [49] (see also refs. [9,56]); (*ii*) the maximum age of about six million years for the lacustrine radiation of the second Tanganyikan clade including *S. dhonti, S. petricola, S. polli *and *S. sp. nov.*, based on the age of the truly lacustrine habitat in Lake Tanganyika [46-48] (see also refs. [9,22,64]) (Note that we did not use the first Tanganyikan clade because of the apparently deviating evolutionary rate in that clade and the possibility that *S. granulosus *might represent a separate lineage that existed before the formation of the lacustrine habitat in the Lake Tanganyika basin); and (*iii*) the minimum age of about 20 million years for the most recent common ancestor (MRCA) of the East African clade of *Synodontis*, based on the oldest *Synodontis *fossils in that area [41-45]. We note that these East African *Synodontis *fossils from the Early Miocene have not been assigned to extant species (see *e.g.*, ref. [45]), which is difficult due to the diversity of the family and the fact that the criteria used for the identification of extant species are not applicable to the fossil specimens [78]. However, because these *Synodontis *fossils resemble extant East African species, and because of the rich fossil record since the Miocene, it is highly likely that these specimens belong to the East African clade. The second and third cycle of analyses aimed to corroborate internal calibrations. We, therefore, ran an analysis with a single calibration point at 20 million years for the minimum age of the East African clade, and an analysis, in which we constrained the maximum age for *S. njassae *to one million year and the minimum age for the East African clade to 20 million years.

## Abbreviations

BI, Bayesian inference; ML, maximum likelihood; MP, maximum parsimony; MRCA, most recent common ancestor; mtDNA, mitochondrial DNA; MY, million years; MYA, million years ago; ND6, NADH Dehydrogenase Subunit 6; NJ, neighbor joining; ti, transition mutation; tv, transversion mutation.

## Authors' contributions

SK, CS, EV, AM and WS designed the study and were involved in sampling. SK and WS carried out the molecular work and the analyses. All authors contributed to the preparation of the manuscript. They read and approved the final version.
